# Independent adaptation mechanisms for numerosity and size perception provide evidence against a common sense of magnitude

**DOI:** 10.1038/s41598-018-31893-6

**Published:** 2018-09-11

**Authors:** Giovanni Anobile, David C. Burr, Marika Iaia, Chiara V. Marinelli, Paola Angelelli, Marco Turi

**Affiliations:** 1 Department of Developmental Neuroscience, Stella Maris Scientific Institute, Calambrone Pisa, Italy; 2 Department of Translational Research on New Technologies in Medicines and Surgery, University of Pisa, Pisa, Italy; 3 Department of Neuroscience, Psychology, Pharmacology and Child Health, University of Florence, Florence, Italy; 4 Department of History, Society and Human Studies, Lab. of Applied Psychology and Intervention, University of Salento, Lecce, Italy; 5 IRCSS Santa Lucia, Rome, Italy; 6 Fondazione Stella Maris Mediterraneo, Chiaromonte, Potenza, Italy

## Abstract

How numerical quantity is processed is a central issue for cognition. On the one hand the “number sense theory” claims that numerosity is perceived directly, and may represent an early precursor for acquisition of mathematical skills. On the other, the “theory of magnitude” notes that numerosity correlates with many continuous properties such as size and density, and may therefore not exist as an independent feature, but be part of a more general system of magnitude. In this study we examined interactions in sensitivity between numerosity and size perception. In a group of children, we measured psychophysically two sensory parameters: perceptual adaptation and discrimination thresholds for both size and numerosity. Neither discrimination thresholds nor adaptation strength for numerosity and size correlated across participants. This clear lack of correlation (confirmed by Bayesian analyses) suggests that numerosity and size interference effects are unlikely to reflect a shared sensory representation. We suggest these small interference effects may rather result from top-down phenomena occurring at late decisional levels rather than a primary “sense of magnitude”.

## Introduction

Many properties of objects, such as duration, size, length, density, numerosity and so on, tend to be correlated with each other, carrying congruent information. For example, the slowest queue to get to the cashier will usually also be the more numerous, the longer and the denser. We thus could use any or all these magnitudes to select the fastest supermarket line. Are these different magnitudes processed independently, or do they share mechanisms? – this remains an open and highly debated issue.

More than ten years ago Walsh^1^ proposed his influential ATOM theory (A Theory of Magnitude), suggesting that magnitudes like space, time and numerosity form part of a generalized system, mainly located in the parietal cortex^2^. Walsh’s main goal was to understand why parietal cortex is organized as it is, and his emphasize was on the role of motor actions in establishing a common metric for representing space, time and number. Many subsequent investigations supported this theory, showing that actions shape magnitude perception and vice-versa. For example saccadic eye movements cause compression of perceived time as well as space^3^; motor adaptation distorts numerosity perception^4^; number magnitude influences grip aperture^5^; self-produced hand actions modulate the interactions between auditory time and visual motion direction^6^; voluntary actions shape time perception^7,8^; locomotion impairment (paraplegia) drastically reduces visual sensitivity for perceiving biological motion^9^.

Recently the discussion has moved to whether numerosity can be perceived independently of other magnitudes. This issue becomes particularly relevant as the “number sense” has been hypothesized to act as an early precursor for the later development of mathematical abilities^10,11^. Along these lines, a recent paper^12^ suggested that the “number sense theory” should be dismissed in favor of a more generalized “magnitude sense theory”. The authors suggested that the ability to perceive numerosity is not innate but acquired by learning the correlations between other magnitudes, such as density and area. The “magnitude sense theory” enjoys support from results coming from interfering and “Stroop-like” paradigms^13^ in which participants are asked to judge a stimulus dimension such as dot numerosity, while some other task-irrelevant magnitude is manipulated (such as dot size). The rationale beyond most of these studies is that if two dimensions are independent, the judgment/encoding of the first should be not influenced by variations in the other. In this way many interactions have been discovered. For example, visual stimuli with higher numerosity, larger area or higher luminance are perceived to last longer^14^. Similarly, both numerosity and density of crowded dot textures are overestimated when presented within a larger area^15^. If asked to select which ensemble has more dots, adults are faster and more accurate when the more numerous ensemble is also that containing larger dots. This effect can also act in the opposite direction, with size judgments influenced by numerosity^16^. Interactions between numerosity and other magnitudes have also been demonstrated by electroencephalography^17,18^ and imaging techniques^19^. However, questions remain open: do system interactions preclude the existence of specialized mechanisms? At what processing level do these interactions arise? Although the “magnitude sense theory” postulates a shared sensory representation for numerosity and magnitude, throughout cortical recycling^20^, interference may also arise at late decisional stages, well beyond primary sensory processing. Moreover, many primary perceptual attributes interact with each other while maintaining clear separate representations. For example, visual motion interacts with contrast, colour, form and time^21,22^.

Here we addressed these questions behaviorally, measuring perceptual adaptation in children. Perceptual adaptation is a transitory perceptual distortion after a stimulus has been observed for some time (even a few seconds), a form of short-term plasticity^23–25^. A famous example is the waterfall illusion: after observing the downward moving water, the stationary rocks surrounding the waterfall seem to move in the opposite direction^26,27^. Similarly, numerosity and size are susceptible to adaptation: after observing a large object (size) or a stimulus containing many objects (numerosity) the subsequent object or ensemble will appear smaller or less numerous than it physically is^28–30^. Perceptual adaptation taps into the proprieties of brain circuits, affecting the balance of neural activation. It can be quite specific, with its strength depending on the similarity between adapter and test stimuli^23–25,31^. These peculiarities made perceptual adaptation particularly useful to behaviorally test whether different features (e.g. size and numerosity) activate the same brain circuits.

Taking advantage of this evidence we tested whether numerosity and size are encoded by a shared sensory system, as postulated by the “sense of magnitude theory”. The rationale is simple: if numerosity and object size tap neural mechanisms that are to some extent shared, there should be correlations between the magnitude of adaptation to the classes of stimuli. That is, we predict that those participants showing stronger numerosity adaptation should also be those with stronger size adaptation. The same logic can be applied to discrimination thresholds, which should also be correlated. Lack of correlation speaks against the idea of a single sensory mechanism encoding both features. Moreover, a single sensory system for numerosity and size perception predicts qualitative and quantitative similarity for both adaptation and threshold values. Finally, taking advantage from recent evidence for independent sensitivity developmental trajectories for numerosity and some uncountable magnitudes^32^, we also predict independent developmental trajectories for numerosity and size adaptation. Our sample comprised 8–11 year-old children. This development phase is particularly interesting because it is the time when sensitivity levels for many visual functions including motion coherence, form coherence, covered area, time, density and spatial numerosity approach the adult-like level^33,34^. This period is thus particularly sensitive to detect eventual differences in developmental rate, such as different development trajectories for numerosity and density^35^. This is also the age when diagnoses for learning disorders are usually made with confidence, and where robust deficits in numerosity sensitivity in dyscalculia have been documented.

## Results

### Task reliability

Before the main analyses, the reliability of each tasks were measured with a split-half method modified for psychophysical procedures (see Method for details). Reliability values, reported in Table 1, ranged from 0.55 to 0.8 (average 0.65). These values are very close to levels that have yielded strong correlations^4,35^, giving us confidence that lack of correlation does not stem from poor reliability.

**Table 1:** Psychophysical tasks summary statistics and reliability (split-half).

Parameters	Size	Numerosity
WF baseline	Mean: 0.08	Mean: 0.6
	StDev: 0.05	StDev: 0.53
	Reliability: 0.5 ± 0.14	Reliability: 0.54 ± 0.13
WF after adaptation to ‘low’ stimuli	Mean: 0.07	Mean: 0.38
	StDev: 0.02	StDev: 0.24
	Reliability: 0.62 ± 0.12	Reliability: 0.56 ± 0.12
WF after adaptation to ‘high’ stimuli	Mean: 0.11	Mean: 0.37
	StDev: 0.08	StDev: 0.31
	Reliability: 0.68 ± 0.13	Reliability: 0.55 ± 0.13
Adaptation effect induced by ‘low’ stimuli (%)	Mean: 14.9	Mean: 3.1
	StDev: 11.8	StDev: 35.9
	Reliability: 0.83 ± 0.05	Reliability: 0.67 ± 0.13
Adaptation effect induced by ‘high’ stimuli (%)	Mean: 21.2	Mean: 28.1
	StDev: 10.9	StDev: 22.7
	Reliability: 0.77 ± 0.12	Reliability: 0.62 ± 0.14
Total adaptation effect (%)	Mean: 48.2	Mean: 47.6
	StDev: 22.8	StDev: 37.0
	Reliability: 0.82 ± 0.08	Reliability: 0.6 ± 0.12

### Size and numerosity adaptation effects: group analysis

**Figure 1:**
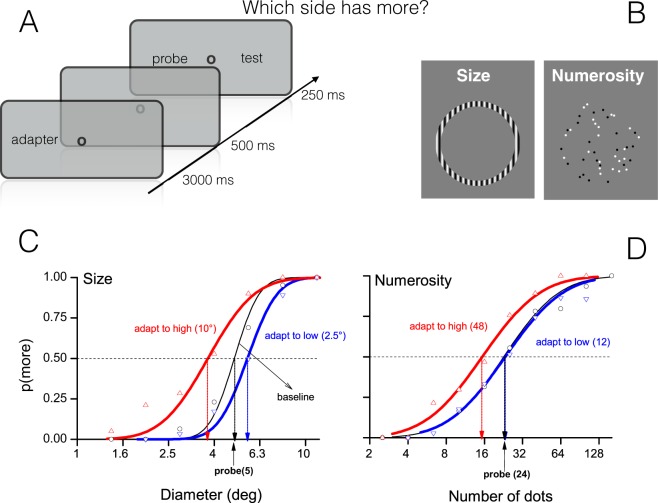
Methods and aggregate psychometric functions. (**A**) Each trial started with a central fixation point followed by the adaptation phase in which the adapting stimulus was displayed on the left of the screen. During this phase, participants fixated the central spot and pressed the space bar when s/he saw it flashing (1/3 of the trials). After this phase a blank pause of 500 ms preceded the test phase in which two stimuli were simultaneously presented for 250 ms on the left and on the right of the central spot. Participants indicated which of the two was perceived as higher/larger. (**B**) Example of stimuli used for the size and numerosity perception tasks. (**C**,**D**) Psychometric curves for the three adaptation levels: baseline (black), adaptation to “low” (half-numerosity or half-size of probe: blue) and adaptation to high (twice probe: red). The curves are generally separated from each other, showing that adaptation occurred. Leftward shifts indicate probe underestimation, rightward overestimation. Vertical arrows point to PSEs values.

Figure 1C,D shows aggregate data together with their fitted psychometric functions. The curves are separated, showing reliable adaptation effects. After adapting to high numerosity or size (48 dots or 10° diameter), the curves are shifted leftward (red) relative to baseline (black), indicating an underestimation of the probe stimulus. Pooled data show that after adaptation to 48 dots a subsequent ensemble of 24 dots was perceived as 15 dots (≈37%). A similar effect (≈30%) was observed for the size task, with a 5° diameter circle perceived as 3.5° after adaptation to a large circle of 10°. Contrarily, rightward shifts indicate overestimation (blue curves). In this condition we found the effect only for size adaptation (≈15%).

**Figure 2:**
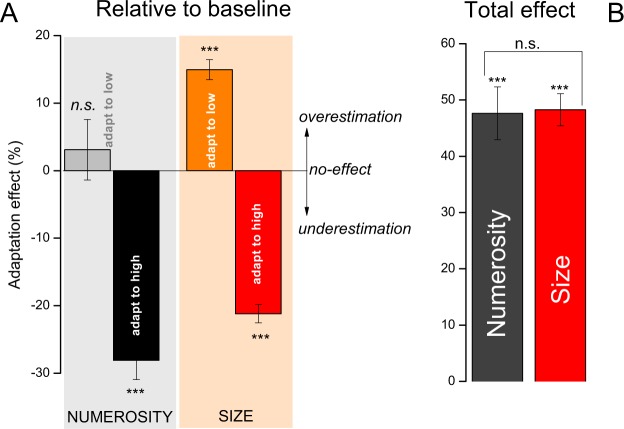
Average adaptation effects. (**A**) Adaptation effects for low and high adapters, compared with baseline (see eq. 1). (**B**) Total adaption effects (normalised distance between PSEs in the two adaptation levels). Error bars are standard errors of the mean. **p < 0.01, ***p < 0.001.

This procedure was separately applied to each participant (average values reported in Table 1). A repeated measure ANOVA with ‘adaptation level’ (2 levels: high and low) and ‘task’ (2 levels: numerosity and size) as factors reveals a significant interaction, meaning that adaptation levels impact differently on size and numerosity tasks (F(1,63) = 5.59, p = 0.02). As mentioned before size but not numerosity was affected by the adaptation to low adapters (the asymmetry is clearly visible in both Figs. 1C,D and 2). Post-hoc t-tests reveal that all but adaptation to low numerosity differs from zero-effect (numerosity: t_63_ = 10.1, p < 0.001; t_63_ = 0.18, p = 0.85; t_63_ = 9.87, p < 0.001 for total effect, low and high; size: t_63_ = 16.8 p < 0.001; t_63_ = 10 p < 0.001; t_63_ = 15.5 p < 0.001, same condition order). Interestingly, total effects do not differ between size and numerosity (t_63_ = 0.135 p = 0.89, Fig. 2B), and for both tasks high adapters (double probe) elicited stronger after-effects than low adapters (half probe): t_63_ = 9.6 p < 0.01 and t_63_ = 4.57 p < 0.01 for numerosity and size.

### Correlation between numerosity and size adaptation effects

**Figure 3:**
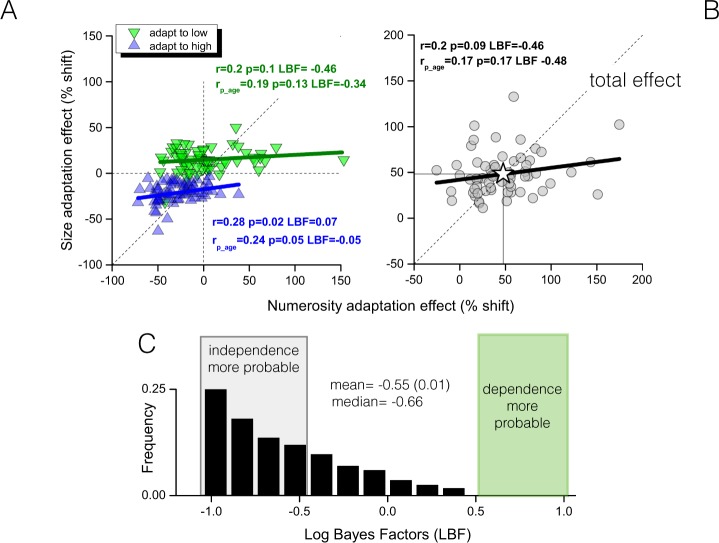
Correlations between adaptation effects. Size against numerosity adaptation effects (**A**) separated into the two adaptation levels (inverted green triangles and upright blue triangles for adaptation to low and high adapters) and (**B**) for total effect. In all cases those participants (single symbols) having higher size adaptation are not those also showing stronger numerosity adaptation. (**C**) Overall frequency distribution of Log Bayes Factors measured for zero-order correlations reveals that is much more probable that the correlation does not exists (LBF < 0.5). Lines are best fitting linear regression fits, “r” reports Pearson zero-order correlation coefficients, “r_p_” reports Pearson partial correlation with chronological age controlled for, LBF are Log Bayes factors.

If size and numerosity perception share some brain mechanisms, we expect to find positive correlations between their adaptation effects. Figure 3 shows size adaptation effects against those measured for numerosity. Panel A shows adaption to low and high adapters separately (green and blue symbols respectively), panel B the total effect (sum of low and high). In all cases correlations were very low, statistically insignificant (p > 0.05), and with Log Bayes Factors clearly pointing to the independence of the two measures (LBF = −0.46, 0.07 and −0.46 for adapt to low, high and total effect respectively).

**Figure 4:**
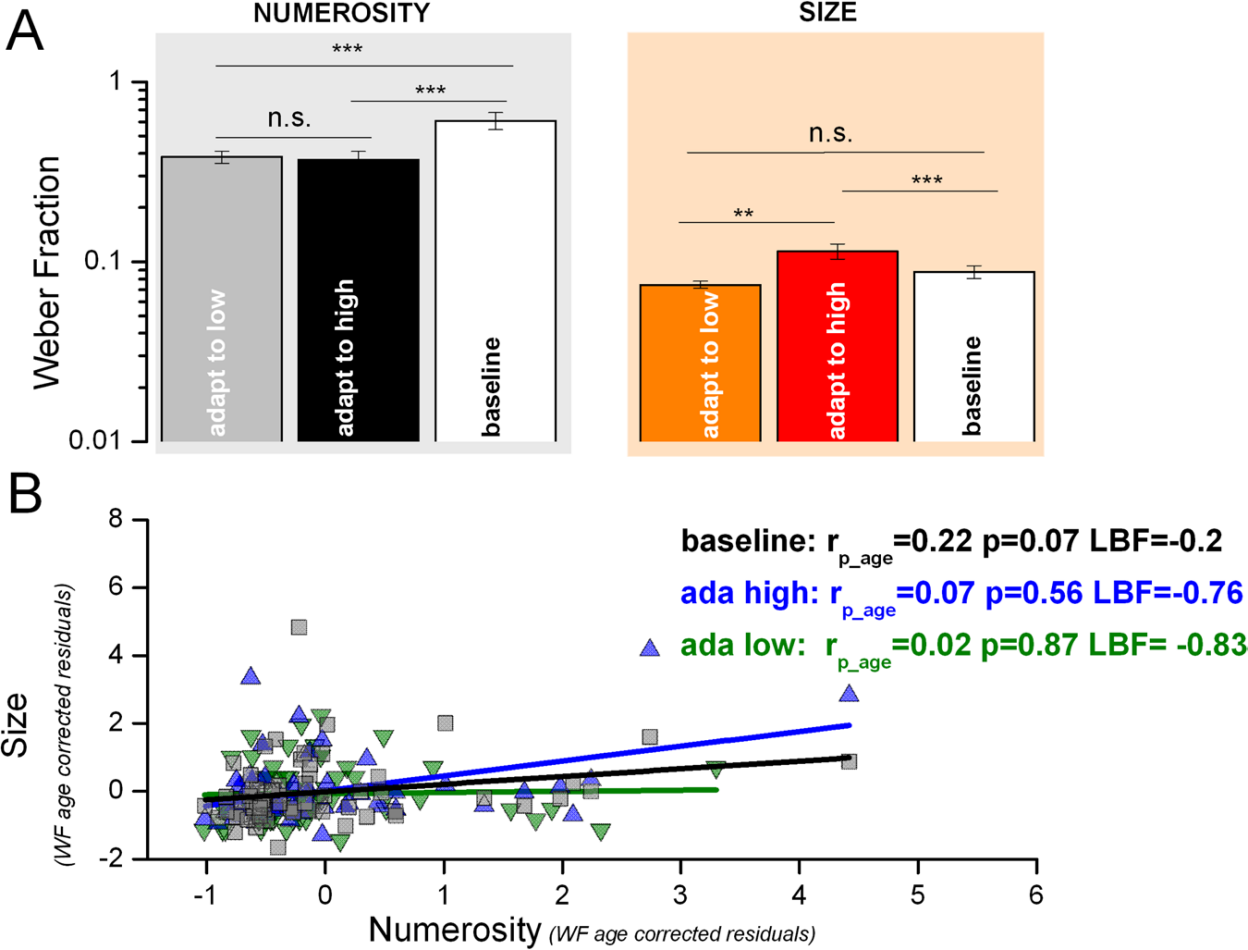
Discrimination thresholds between tasks. (**A**) Weber Fraction averaged across participants. (**B**) Partial correlations between Weber Fractions measured in the size and numerosity discrimination tasks separated by adaptation levels (baseline = black squares, adaptation to low adapter = inverted green triangles and adaptation to high adapter = upright blue triangles).

Figure 3C examines further potential correlations by a bootstrap procedure. It shows the distribution of LBFs (pooling together the three adaptation measures) obtained by a bootstrapping the data. On each of 1000 iterations, for each participant and separately for numerosity and size, we resampled the raw data, fitted psychometric functions to extract the PSEs, measured the adaptation effects, correlated between each other (separately for the three conditions), and measured the three corresponding LBF values (as in Fig. 4A,B). At the end of the procedure, the three frequency distributions of 1000 LBF each were pooled together. Even without controlling for covariates, LBF values were always low: they never signalled substantial existence of a true correlation (LBF >0.5), and for 96% of bootstraps they signalled substantial evidence for a lack of correlation (LBF <−0.5). When chronological age was kept constant, correlations decreased even more (r_p_age_ in the figure), with size adaptation strength explaining only a very small portion of the variance in the numerosity adaptation strength (5%, 4% and 2% in case of adaptation to high, low and for the full effect respectively), and low LBF values (high = −0.34, low = −0.05 and full effect −0.48). As a benchmark of how strong two adaptation tasks activating the same neural circuit might correlate, for each task, we used the correlation coefficients given by correlating the adaptation effects elicited by the two adapter levels (high and low). As expected, these within-task correlations (also correcting for age) were robust (r_p_ numerosity = 0.64 and r_p_ size = 0.47, both p < 0.001). This indicates that those children showing higher adaptation susceptibility for high adapters are also those having stronger adaptation for low adapters. More importantly, this also suggests that when two adaptation tasks likely activate the same mechanism, much higher correlations then those found between tasks are expected (Fig. 3A).

### Size and numerosity discrimination thresholds

If size and numerosity perception share the same brain areas or perceptual mechanisms, we might expect similar and correlated discrimination thresholds levels (Weber Fraction, WF hereafter). Figure 4A shows average WF for numerosity and size tasks. A repeated measures ANOVA with ‘task’ (2 levels: numerosity and size) and ‘adaptation’ (3 levels: high, low, baseline) reveals a main effect of task, with numerosity discrimination having overall higher WF than size (F_(1,63)_ = 114, p < 0.001). Post-hoc t-tests confirmed higher WF for numerosity in all the conditions (t_(63)_ = 9.9, t_(63)_ = 6.35, t_(63)_ = 8.01 all p < 0.001 for adaptation to low, high and baseline respectively). Also the interaction was significant (F_(1,63)_ = 11.6, p < 0.001) indicating that WF for numerosity and size discrimination differs across adaptation levels. Indeed, post-hoc t-test revels that WF for numerosity was higher in the baseline than in both adaptation conditions (t_(63)_ = 3.7 p < 0.001; t_(63)_ = 3.8 p < 0.001 for low and high respectively). However, for size discrimination, baseline average WF was significantly lower than that measured in the adaptation to high (t_(63)_ = 2.78 p = 0.007) but not to low conditions (t_(63)_ = 1.7 p = 0.09). Finally, WF in the two adaptation levels was similar for numerosity (t_(63)_ = 0.29 p = 0.76), while for size adapting to large stimuli leads to higher WF than adapting to small (t_(63)_ = 3.54 p = 0.001). We then looked at correlations between WFs across and within tasks. As all WFs decrease with age in all conditions (numerosity: r = −0.3 p = 0.009, r = −0.16 p = 0.09, r = −0.15 p = 0.12 for low, high and baseline conditions respectively; size: r = −0.11 p = 0.19, r = −0.18 p = 0.07, r = −0.26 p = 0.02, same condition order, one-tail p-values), the subsequent analysis was performed keeping chronological age constant. Figure 4B shows that WF measured in the two tasks did not significantly correlate in any condition (all p > 0.05), with LBF sustaining the null hypothesis (independence). Importantly, as expected inter-task correlations (partial, corrected for age) were all positive and reasonably strong, meaning that those participants having higher WF in the baseline condition were also those having higher WF in the adaptation conditions. This was generally true for both numerosity and size tasks (numerosity: r = 0.38, p = 0.002; r = 0.39, p = 0.002; size: r = 0.16, p = 0.19, r = 0.49, p < 0.001; for adaptation to low and high levels respectively). Again, these correlation values give an idea of how much two experimental conditions are likely to be stimulating the same perceptual system should be (r≈0.35 on average). Overall these results indicate that size perception is much less noisy than numerosity and that their noise levels did not correlates across participants; again suggesting that these visual features are probably processed by two different perceptual systems.

### Relationship between sensory thresholds and adaptation effects

Both sensory thresholds and adaptation and are thought to reflect sensory processing but their relationship is not clear. In order to test this, we measured by hierarchical regression how much variance in the baseline thresholds (dependent variable) was explained by the adaptation strengths (independent variables), separately for numerosity and size, while controlling for age (entered in the first stage). Adaptation effects to low and high stimuli were entered together as a block in the second stage. Both for numerosity and size, adaptation effects explain a significant portion of variance in the baseline thresholds values, suggesting that these two measures share some components (numerosity: R_change_ = 15% p = 0.007; size: R_change_ = 16% p = 0.003). We then looked at the direction of relationships by partial correlations (controlling for age). Interestingly, correlation coefficients were generally negative, meaning that higher WFs in the baseline condition were associated with more underestimation induced by adaptation to high magnitude adapters (numerosity: r_p_ = −0.37, p = 0.003; size: r_p_ = −0.43, p = 0.001). For low adapters the results were less clear with only numerosity adaptation correlating with WFs (numerosity: r_p_ = −0.33, p = 0.008; size: r_p_ = −0.20, p = 0.1 for low adapters). The direction of this last correlation indicates that participants with higher Wfs were also those with higher numerosity underestimation induced by low adapters. Regarding the total effect, positive correlations were expected because in this case the effect was measured as the overestimation induced by adapting to low compared to the PSEs measured after adapting to high stimuli. In this case we found positive correlations but only that for the size task reach the significance level (numerosity: r_p_ = 0.07, p = 0.56 and size: r_p_ = 0.41, p = 0.001).

### Developmental trajectories of size and numerosity adaptation

**Figure 5:**
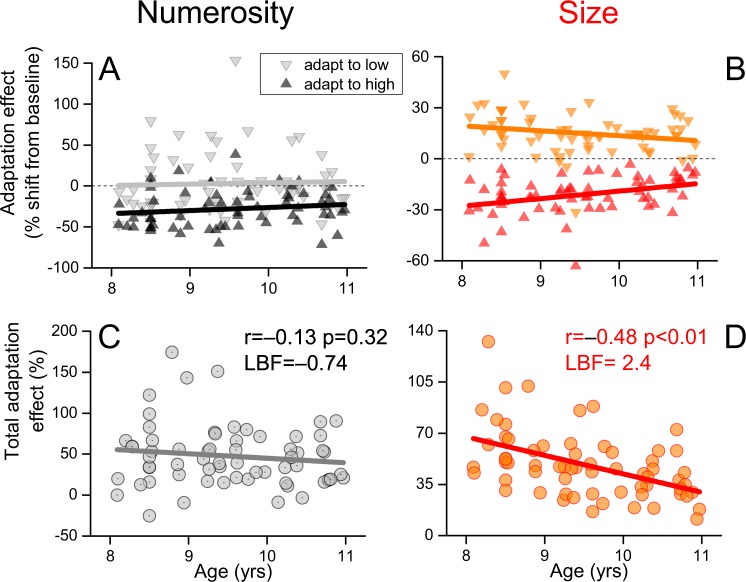
Developmental trajectories. Numerosity and size adaptation as a function of age, separated for adaptation levels (**A**,**B**) and for total effect (**C**,**D**). Size but not numerosity adaptation significantly decreases with age. Lines reflect best fitting linear regression fits and r reports Pearson coefficients.

Figure 5 shows adaptation effects as a function of chronological age. From inspection it is clear that strength of size adaptation decreases with age, while that for numerosity task did not. Correlations for size: r = −0.21, p = 0.04, LBF = −0.4; r = −0.35 p = 0.002, LBF = 0.72; r = −0.48, p < 0.01, LBF = 2.46) for low, high adapters and total effect) for numerosity: r = 0.04, p = 0.37, LBF = −0.98; r = 0.14, p = 0.12, LBF = −0.74; r = −0.13, p = 0.32, LBF = −0.78 (for low, high adapters and total effect).

We measured correlation differences on the overall effects (panels C and D) by a bootstrap method. On each of 1000 iterations we sampled (with replacement) the data and measured the correlations (Pearson-r) between age and adaptation effect separately for size and numerosity tasks. The proportion of time that numerosity r-coefficients were lower than those for size was the p-value (sign-test, one-tail). Averages r-coefficients were −0.48 ± 0.08 and −0.12 ± 0.11 for size and numerosity, with their difference highly statistically significant (p = 0.001). As low inter-subject variability may obscure correlations, to check whether the different dependence on age was due to a higher degree of inter subject variability in the size task, we ran a bootstrap test where on each iteration (1000) we measured the group standard deviation for size and numerosity total adaptation effects separately. In this case p-values were obtained doubling the proportion of time that the standard deviation in the size task was lower than that for the numerosity task (sign-test, two-tail). Interestingly, adaptation effects in size were less variable than those in numerosity (average standard deviations were 22 ± 2.7 and 36.8 ± 4.6 for size and numerosity respectively, p = 0.004), so that could not explain the lack of correlation between numerosity and age. Following a recent technique^32^, we then tested whether the developmental trend of size adaptation depends on development of the numerosity system. We thus ran a partial correlation between size adaptation (total effect) and age, partialling out the numerosity adaptation effect (total) and discrimination thresholds (WF for all the three adaptation levels were used together, 4 covariates in total). The results show that even after partialization, size adaptation significantly decreases with age (r_p_ = −0.46, p < 0.001). Overall, these results indicate that numerosity and size adaptation differ in their dependence on age, with size decreasing much more than and independently from numerosity.

## Discussion

In this study we tested whether numerosity and size are encoded by a shared sensory mechanism, as postulated by the recent “sense of magnitude” theory^12,20^. In brief, the results show that normalized size thresholds (Weber fractions) are much lower than those for numerosity, and individual differences do not correlate over the two tasks, neither for adaptation strengths nor for threshold values. Adaptation to size but not to numerosity was found to be bidirectional and only adaptation to size changes with age.

Lack of correlation does not necessarily mean true independence. For example, high experimental error could mask a real correlation. However, that is highly unlikely in our case, as all the individual tasks have high reliability (measured by a bootstrap technique), similar to the reliability levels of previous published studies (≈0.65: Table 1) that led to strong correlations^35,36^. Furthermore, there are strong correlations between different measurements within each task, with baseline thresholds correlating well with thresholds after high and low adaptation, and adaptation strength caused by high magnitude stimuli correlating with that elicited by lower magnitude stimuli.

All this evidence suggests separate mechanisms for numerosity and size perception, inconsistent with the recently proposed “sense of magnitude” theory^12^, which proposes that the capacity to perceive numerosity might be not be innate but acquired by learning the correlations between numerosity and continuous features such as area, size, density and so on. The domain-specific idea of a “number sense” gives way to a domain general “magnitude sense”. In a subsequent paper^20^, they specifically focused on the role of size perception on the foundation of numerical processing, suggesting that it might play a crucial role, even sharing brain representations. Indeed, many interactions between those two stimuli features pointed to the existence of a shared mechanism. For example, it is easier to say that a digit is numerical higher than another when also its physical size (font) is congruently larger (3 5 *versus* 3 5); the same occurs when comparing numerosities, with dot physical size influencing numerosity judgments and vice-versa (for a meta analyses on size congruity effects see also^37^). Henik and collaborators^20^ also clearly specify that interactions may occur at a sensory level through cortical recycling: “routines and neural structures built for size judgments were made available to other systems, through evolution, allowing for the development of an exact numerical system. Noncountable representations and the ability to perceive and evaluate sizes or amounts were essential for such development”. Interestingly, cortical recycling is the mechanism that has been recently hypothesized to bind space and number^38^: it has been shown that numerosity adaptation to dot patches was influenced by the motion direction (space) of the adapter dot patch. Following a similar rationale, a recent work showed that size adaptation does not affect perceived numerosity: adapting to a large (or small) disk (size adaptation) leaves subsequent numerosity estimation of sparse dots arrays almost unaffected in adults^39^. Our data fit well with these studies, showing independence between size and numerosity individual differences for both adaptation strengths and thresholds, in children. The correlational approach used here also unveiled an unexpectedly large inter-subject variability in perceptual adaptation susceptibility in children. This mirrors the large variability usually described for sensitivities^11^, and raises the question of how much of this variability may be shared by other perceptual/cognitive processes. Moreover, as those children more susceptible to numerosity adaptation were not more susceptible to size adaptation, it seems that perceptual plasticity maintains a certain level of autonomy for different features.

There is also evidence suggesting separate mechanisms for numerosity and size from fMRI adaptation studies. Castaldi *et al*.^40^ found that numerosity adaptation changed activity in IPS, leaving earlier visual cortices (V1) unchanged. On the other hand, Pooresmaeili, *et al*.^30^ showed that size adaption acts as early as V1. While the theory of Leibvoich *et al*. clearly suggests a shared sensory mechanism for numerosity and size perception^12,20^, it is less clear exactly what the ATOM theory predicts for early primary sensory processing interactions. The ATOM theory is mainly concerned with how and why the parietal cortex is organized as it is, particularly the role of sensorimotor integration. However, even if numerosity adaptation acts primarily on parietal cortex, while that for size on V1, passive encoding of both features activates both areas^41–43^.

While correlation does not imply causation, focusing of individual differences can reveal interesting links, or lack of expected functional connections. One example is the classical dichotomy between the dorsal and ventral streams^44^. Performance on dorsal tasks correlate with each other, suggesting a shared sources of variance, but much less with those encoded by ventral areas^33,45^. Similarly, Halberda, *et al*.^11^ showed that a significant portion of inter-subject variance in math abilities was explained by numerosity thresholds, suggesting a shared system for symbolic and non-symbolic numbers. The same logic has been applied to disentangle perceptual systems underling the encoding of numerosity and size. For example Agrillo, *et al*.^46^ found that adult thresholds for length and numerosity did not correlate between each other, suggesting separate mechanisms. Starr and Brannon^47^, however, found that thresholds for numerosity and line length comparison were significantly correlated in both children and adults. Similarly, a recent study found null correlations between object size and numerosity discrimination thresholds in both children and adults^35^. Lourenco, however, found a significant link between numerosity and cumulative area estimations^48^.

Taking together this and other evidence suggests that numerosity and size might be subserved by at least partially separate systems. Besides correlational studies, training protocols also showed independence between numerosity and size. Along these lines, Piazza, *et al*.^49^ showed that exposing unschooled indigenous people to mathematical knowledge improves sensitivity for numerosity but not for object size. Ferrigno, *et al*.^50^ trained preschool children, schooled and unschooled adults, as well as monkeys to associate and categorize dot arrays into two categories, small or large. Number and cumulative area were completely correlated during the training. After training, participants were exposed to stimuli where number and cumulative area were uncorrelated. The results show that categorization judgments on these test trials followed numerosity and not area, despite differences in age, species and education. In other words, subjects are universally predisposed to base their judgments on number as opposed to the alternatives. Similarly Cicchini, *et al*.^51^ recently addressed this issue by leaving density, number and area of patches free to vary and asked participants to judge which one of three stimuli (two were identical) was the odd-one-out, never mentioning numerosity. Also in this case, participants spontaneously used numerosity rather than size or density as decisional criteria. Even children as young as six years of age also spontaneously encode numerosity^52^. Our data fit well with the idea that humans can represent numerosity regardless other stimuli dimension, such as size.

Differences in developmental trend also can add information on this topic. Odic^34^ recently showed that sensitivity to numerosity discrimination continues to improve as a function of age, even when sensitivity for time, area, length and density were controlled for by partial correlations. Similarly, we found here that numerosity and size adaptation strengths evolve quite differently. Between 8 and 11 years, numerosity adaptation strength remained almost stable, suggesting that it was already saturated, while susceptibility to size adaptation robustly decreased with age. It should be noted, however, that the ATOM theory predicts that continuous magnitudes (like space, time and number) originate from a single metric, but leaves open the possibility for different developmental trajectories. Indeed Walsh^53^ suggested that different magnitudes could develop differently due to different levels of relevance, difficulty, feedback, language etc. However, different rates do not imply independence, as the systems could still share variance. Here, similarly to Odic^34^ we found that size adaptation continues to change with age even while simultaneously controlling for numerosity thresholds and total adaptation level (partial-r between size adaptation strength and age r_p_ = − 0.46, p < 0.001 with age, accounting for 21% of variance). These results together suggest again different systems for size and numerosity perception.

Finally, another clear difference between numerosity and size adaptations comes from the directionality of the effect: for size task both overestimation and underestimation occur after adaptation to low and high adapters, while numerosity was (on average) affected only by high adapters. This last result may seem in contrast with the adult literature where numerosity adaptation bidirectionality was well established^4,28,29,54^. We do not have a definitive explanation for this developmental difference but could speculate that it may relate to coarser receptive fields for low than high numbers, so adaptation to low numbers may spread over a larger portion of visual space and affect both test and probe stimuli, washing itself out. However, whatever the reason, the asymmetry between size and numerosity again points to structurally different sensory mechanisms governing their encoding.

Although these results generally suggest that numerosity and size might not be encoded by the same system, Leibovich and colleagues have brought much evidence to the contrary listing many studies showing behavioural interactions. Our opinion is that this apparent contradiction depends on the interactions measured by that particular behavioural method. In our case we have used two sensory parameters (thresholds and adaptation), measuring characteristics strictly related to the nature of the perceptual system involved in the encoding of that particular feature. In this study, we measured perceptual adaptation and sensitivity in two separate tasks rather than pitting numerosity perception against other naturally correlated features. Indeed, from an evolutionary perspective all the interactions documented between numerosity and other features may be adaptive for an environment where numerosity is inevitably correlated with other continuous features (such as size). Thus when numerosity is pitted against those features, interactions might emerge as drops in performance, or even as interactions in brain activation level. In line with this, some studies have highlighted the role of cognitive control necessary to inhibit irrelevant dimensions that are incongruent with numerosity^55–58^. In other words, the inconsistencies found so far may reflect the level of processing probed by the task. Even within interfering paradigms, task-related inconsistencies emerge, clearly speaking against a bottom-up level of interaction involving shared sensory processing. A clear example is the study of Leibovich and colleagues^59^ who recently showed that physical size affects number but only in some specific conditions: only for discrimination (not-estimation) of non-symbolic numerosity (not Arabic digits), above the subitizing range (>4), for dots presented in non-canonical format (e.g. not for dice like pattern). Moreover, numerosity verbal estimation or mapping of small dots is often overestimated and numerosity of large dots underestimated^60–62^, while the opposite trend has been documented for comparison tasks^16,63^. Moreover, when perceptual discriminability was matched, overall dot size was found to not interfere with numerosity perception^64^. To summarize we found that neither perceptual adaptation strength nor thresholds correlated between numerosity and size discrimination tasks. Moreover, we also found independent developmental trajectories for the two magnitudes. These data provide evidence that numerosity and size are not encoded by a fully shared sensory mechanism, and that previous behavioural interactions between numerosity and size are likely to reflect cognitive, rather than sensory interactions. These results, together with other evidence, points to the existence of a specialized “number sense”, rather than a more general “magnitude sense”. As almost all visual features are susceptible to perceptual adaptation (e.g. density, time, brightness, length) our methodology can be applied to virtually all of them.

## Methods

### Participants

64 children (8.0–10.9 years old, mean 9.5), were included in this study. One child was eliminated from the dataset because s/he was absent during the second data collection session.

Children were recruited from local schools of Lecce. All participants scored above the 10th percentile (mean 63.89 std: 27.19) on Raven’s Coloured Progressive Matrices (CPM, according to data from^65^.

The parents were informed of the screening activities and authorized their child’s participation by signing the appropriate informed consent, which was set up and collected by the University of Salento. The study was conducted according to the principles of the Helsinki Declaration and was approved by the school authorities and from the committee of the University of Salento.

### Data analysis

Points of subjective equality (PSE) and discrimination thresholds (Weber Fraction) were measured separately for each participant, task and condition. Adaptation effects were measured for high and low adapters as PSEs percentage shift from baseline (eq. 1).

1 $${Adaptationeffect}=100\times (\frac{{PSEadapt}-{PSEbaseline}}{{PSEbaseline}})$$

The “*total effect”* instead refers to the distance between PSEs measured in the two adaptation level (high and low stimuli) without considering the baseline (eq. 2).

2 $${Totaladaptationeffect}=100\times (\frac{{PSEadapttolow}-{PSEadapttohigh}}{{PSEadapttohigh}})$$

Data were analysed by repeated measures ANOVAs, hierarchical regressions, t-test, Pearson correlations (zero-order and partials) and non-parametric bootstrap. Significance of correlations were calculated by p-values and also by Bayes Factor^66^. Bayes factor is the ratio of the likelihood probabilities of the two models H1/H0, where H1 is the likelihood of a correlation between the two variables, and H0 the likelihood that the correlation does not exist. By convention, a Log Bayes Factor (LBF) > 0.5 it is considered substantial evidence in favour of the existence of the correlation, and LBF < −0.5 substantial evidence in favour of it not existing. Task reliability of the psychophysical tasks was measured (see Table 1) using a split-half “sample-with-replacement” (non-parametric) bootstrap technique suitable for reliability of measures extracted form psychometric functions^35,36^. For each participant, separately for each adaptation condition (low, high, baseline) and task (size, numerosity), we fitted two separate psychometric functions taking data from a random sample (as large as the data set, sampled with replacement from the data set), from which we calculated WFs. We computed the correlation between those two estimates of WF for all participants, and reiterated the process 1000 times, to yield mean and standard error estimates of reliability. We employed the same procedure for adaptation effects but on each iteration measured adaptation effects as described before. Data were analysed with R, Matlab and SPSS 20.0.

### Numerosity adaptation task

Stimuli were patches of dots presented on either side of a central fixation point (Fig. 1B). Dots were 0.25 diameter, half-white and half-black (to balance luminance), presented at 80% contrast on a grey background of 40 cd/m2. They were constrained to fall within a virtual circle of 10° diameter, centred at 12° eccentricity. The numerosity of the probe stimulus (on the left) was 24, while the test (on the right) adaptively changed following a QUEST algorithm (Adaptive Staircase^67^). During the adaptation phase the adaptor consisted of a patch of dots with a numerosity that could be 0 (baseline, no dots), 12 (adapt to low, half the probe) or 48 (adapt to high, twice the probe). The three adaptation conditions were separately tested and each session started with 3000 ms of the adaptor presented on the left of central fixation point, then 500 ms after the adaptor disappeared the test and probe were simultaneously presented for 250 ms. Participants indicated by appropriate key-press the side of the screen with more dots. All participants performed 1 session of 45 trials for each adaptation condition (135 trials in total). The proportion of trials where the test appeared more numerous than the probe was plotted against the test numerosity (on log axis), and fitted with cumulative Gaussian error functions (Fig. 2). The 50% point of the error functions estimates the point of subjective equality (PSE), and the difference in numerosity between the 50% and 75% points gives the just notable difference (JND), which was used to estimate Weber Fractions (JND/PSE). To sustain attention, during the adaptation phase a colour change-detection task was placed. Subjects were asked to quickly report any change in colour (black/white) of the fixation point by pressing the spacebar (1:3 of trials).

### Size adaptation task

All procedures were identical to the numerosity task, but here stimuli were luminance-modulated sinusoidal gratings windowed within an annulus. Spatial frequency was 2 c/deg, and Michelson contrast 90% (Fig. 1B). After stimulus presentation, a 100 ms full-screen random noise mask was displayed to annul possible afterimages. The diameter of the probe stimulus was 5°. Adapter stimuli comprised an annulus with a diameter that could be 2.5° (half the probe size: adapt to low) or 10° (twice the probe: adapt to high).
